# Highly Accurate Chimeric Proteins for the Serological Diagnosis of Chronic Chagas Disease: A Latent Class Analysis

**DOI:** 10.4269/ajtmh.17-0727

**Published:** 2018-09-17

**Authors:** Fred Luciano Neves Santos, Ana Clara Paixão Campos, Leila Denise Alves Ferreira Amorim, Edimilson Domingos Silva, Nilson Ivo Tonin Zanchin, Paola Alejandra Fiorani Celedon, Rodrigo Pimenta Del-Rei, Marco Aurélio Krieger, Yara Miranda Gomes

**Affiliations:** 1 Gonçalo Moniz Institute, Oswaldo Cruz Foundation-Bahia, Salvador, Brazil;; 2 Fio-Chagas, Oswaldo Cruz Foundation, Rio de Janeiro, Brazil;; 3 Department of Statistics, Institute of Mathematics and Statistics, Federal University of Bahia, Salvador, Brazil;; 4 Institute of Technology in Immunobiologicals, Bio-Manguinhos, Oswaldo Cruz Foundation-Rio de Janeiro, Rio de Janeiro, Brazil;; 5 Carlos Chagas Institute, Oswaldo Cruz Foundation-Paraná, Curitiba, Brazil;; 6 Molecular Biology Institute of Paraná (IBMP), Curitiba, Brazil;; 7 Aggeu Magalhães Institute, Oswaldo Cruz Foundation-Pernambuco, Recife, Brazil

## Abstract

The existence of an imperfect reference standard presents complications when evaluating the unbiased performance of novel diagnostic techniques. This is especially true in the absence of a gold standard, as is the case in chronic Chagas disease (CD) diagnosis. To circumvent this constraint, we elected to use latent class analysis (LCA). Previously, our group demonstrated the high performance of four *Trypanosoma cruzi*–chimeric proteins (Molecular Biology Institute of Paraná [IBMP]-8.1, -8.2, -8.3, and -8.4) for CD diagnosis using several distinct immunoassays. Although commercial tests had previously been established as a reference standard, the diagnostic performance of these chimeric antigens could present bias because these tests fail to produce 100% accurate results. Thus, we used LCA to assess the performance of these IBMP chimeric antigens in chronic CD diagnosis. Using the LCA model as a gold standard, sensitivity and specificity values ranged from 93.5% to 99.4% and 99.6% to 100%, respectively. The accuracy values were 96.2% for IBMP-8.2, approximately 98% for IBMP-8.1 and IBMP-8.3, and nearly 100% for IBMP-8.4. For IBMP-8.1 and IBMP-8.2, higher positive predictive values were associated with increases in hypothetical prevalence. Similarly, higher hypothetical prevalence resulted in lower negative predictive values for IBMP-8.1, IBMP-8.2, and IBMP-8.3. In addition, samples with serodiscordant results from commercial serological tests were analyzed using LCA. Molecular Biology Institute of Paraná -8.1 demonstrated potential for use in confirmatory testing with regard to samples with inconsistent results. Moreover, our findings further confirmed the remarkable performance of the IBMP-8.4 antigen to diagnose chronic CD in both endemic and non-endemic areas.

## INTRODUCTION

Chagas disease (CD), a life-threatening condition arising from the hemoflagellated protozoan *Trypanosoma cruzi*, represents a major health problem in Latin America.^1^ This disease affects more than five million people in 21 Latin American countries.^2^ In Brazil alone, 4.6 million people were estimated to be infected in 2014.^3^ During recent decades, an increasing number of cases in the non-endemic countries of North America,^4^ Europe,^5^ Asia,^6^ and Oceania^7,8^ have been described. Despite successes in vector eradication and blood donor–screening programs funded by large-scale multinational initiatives,^9^ the incidence of CD continues to increase in areas that lack control measures. Considering the fact that CD is characterized by a prolonged asymptomatic phase and that most affected individuals reside in peri-urban slums or rural areas, improvements in measures to control and prevent CD must be made. Because most chagasic (Ch) individuals in the ongoing chronic phase are never tested and remain unaware of their condition, it is essential to develop accurate CD diagnostic tools for use both in routine health services and at blood centers. Laboratory diagnostic techniques are dependent on illness stage and clinical symptoms. During the acute phase, direct microscopic observation of trypomastigote forms is the preferred method to affirm diagnosis as a result of high parasite load in the blood. In this phase, indirect serological methodologies present low sensitivity due to a delayed humoral immune response.^10^ Symptoms present rarely or are unspecific, including fever and an occasional inflammatory reaction at the infection site.

Conversely, chronic CD is characterized by a substantial decrease in parasitemia and the presence of several specific antibodies (immunoglobulin G) directed against the pathogen.^11^ Despite low parasite levels, approximately 30% of infected individuals develop cardiac and gastrointestinal manifestations.^12^ Under these circumstances, direct microscopic observation fails, whereas indirect serological methodologies have proven highly sensitive. Several serological methods are available for CD diagnosis,^13–17^ with enzyme-linked immunosorbent assays (ELISAs) being the most widely used. However, the performance of these assays varies greatly because of the antigen preparations used to detect the anti–*T. cruzi* antibodies, in addition to genetic heterogeneity among circulating strains.^16,18^ Because no reference standard is currently available for CD, the World Health Organization has advocated the use of two tests in parallel for serodiagnostic verification. Under these circumstances, the diagnostic accuracy of novel methodologies cannot be evaluated in an unbiased manner, because the comparison of results implies the use of an imperfect reference standard. A strategy to circumvent this limitation entails the use of latent class modeling, which assumes that although the true disease status remains unknown, the available methods of evaluation do offer an approximation of the real state of disease. Accordingly, the probability of a given combination of test results yields a latent class status, that is, disease status.^19^ Hence, latent class analysis (LCA) is capable of providing an estimation of disease status in cases in which a true gold standard is lacking.

Recently, our group assessed the adoption of recombinant chimeric antigens comprising repetitive fragments of antigenic *T. cruzi* parasite proteins for the detection of specific antibodies using distinct immunoassays.^20–23^ Although the use of two commercial tests had been previously established as a reference standard,^16^ the diagnostic performance of the present chimeric antigens could be biased due to shortcomings in the accuracy of these commercial tests.^24^ Considering this scenario, we endeavored to evaluate the diagnostic performance of four Molecular Biology Institute of Paraná (IBMP) chimeric antigens using LCA in the serodiagnosis of chronic CD.

## Materials and Methods

### Ethics statement.

The use of anonymized sera samples in this study was approved by the Aggeu Magalhães Research Center Institutional Review Board (CAAE: 15812213.8.0000.5190; FIOCRUZ, Recife, Brazil). Samples were obtained in coordination with routine public health screening for CD conducted by “Dr. Milton Bezerra Sobral” Public Health Central Laboratory-Pernambuco, Hemope Foundation-PE, the CD Reference Laboratory (FIOCRUZ-PE), and the Laboratory for Research on CD from the Federal University of Goiás (Goiás, Brazil).

### Synthetic gene acquisition, protein expression, and purification.

Synthetic genes encoding *T. cruzi* chimeric proteins, denominated IBMP-8.1, IBMP-8.2, IBMP-8.3, and IBMP-8.4 (Table 1), were obtained from a commercial supplier (GenScript, Piscataway, NJ) and subcloned into the pET28a vector.^21^ Isopropyl β-D-1-thiogalactopyranoside was used to induce the expression of IBMP chimeric antigens in BL21-Star (DE3) under suboptimal culturing conditions. Proteins were first purified by both affinity and ion exchange chromatography, and then quantified using a fluorometric assay.

**Table 1:** Constitution of IBMP chimeras

Chimeric antigen	Sequence name	Amino acid range	GenBamk sequence ID
IBMP-8.1	Trans-sialidase	747–774	XP_820062.1
	60S ribosomal protein L19	218–238	XP_820995.1
	Trans-sialidase	1,435–1,449	XP_813586.1
	Surface antigen 2 (CA-2)	276–297	XP_813516.1
IBMP-8.2	Antigen, partial	13–73	ACM47959.1
	Surface antigen 2 (CA-2)	166–220	XP_818927.1
	Calpain cysteine peptidase	31–97	XP_804989.1
IBMP-8.3	Trans-sialidase	710–754	XP_813237.1
	Flagellar repetitive antigen protein	15–56	AAA30177.1
	60S ribosomal protein L19	236–284	XP_808122.1
	Surface antigen 2 (CA-2)	279–315	XP_813516.1
IBMP-8.4	Shed-acute-phase antigen	681–704	CAA40511.1
	Kinetoplastid membrane protein 11 (KMP-11)	76–92	XP_810488.1
	Trans-sialidase	1,436–1,449	XP_813586.1
	Flagellar repetitive antigen protein	20–47	AAA30177.1
	Trans-sialidase	740–759	XP_820062.1
	Surface antigen 2 (CA-2)	276–298	XP_813516.1
	Flagellar repetitive antigen protein	1–68	AAA30197.1
	60S ribosomal protein L19	218–238	XP_820995.1
	Microtubule-associated protein	421–458	XP_809567.1

IBMP = Molecular Biology Institute of Paraná.

### Sampling and in-house ELISA procedures.

Sample size was estimated with a sensitivity and specificity of 99%, an absolute error of 1.5%, and a 95% confidence interval (CI). Based on these specifications, the minimum number of samples needed to perform this study was 380 sera from non-Ch (NCh) and 380 sera from Ch individuals. In all, 1,121 (526 NCh and 595 Ch) previously collected and anonymized human serum samples were enrolled. These sera were obtained from diverse Brazilian geographic areas, both non-endemic and endemic, and from other endemic countries in Latin America, as well as the United States.^20^ Before analysis, all of these sera were simultaneously reevaluated using commercial ELISA tests, namely, ELISA Chagas III (batch 1F130525; BIOSChile, Ingeniaría Genética S.A., Santiago, Chile), Imuno-ELISA Chagas (batch 14D061; Wama Diagnostica, São Paulo, Brazil), Pathozyme Chagas (Omega Diagnostics, Scotland, United Kingdom), and Gold ELISA Chagas (Rem, São Paulo, Brazil).^16^ In addition, to assess the diagnostic accuracy of the four chimeric proteins with respect to CD, another 105 samples that had previously presented serodiscordant results, or were judged to be inconclusive by one of the tests, were analyzed separately using latent class status as a gold standard. Finally, anti–*T. cruzi* serological testing was performed using in-house ELISA procedures as previously described.^21^ Samples from individuals with leishmaniasis (*N* = 153) were used to assess the cross-reactivity using LCA.

### Statistical analysis.

**Figure 1.**
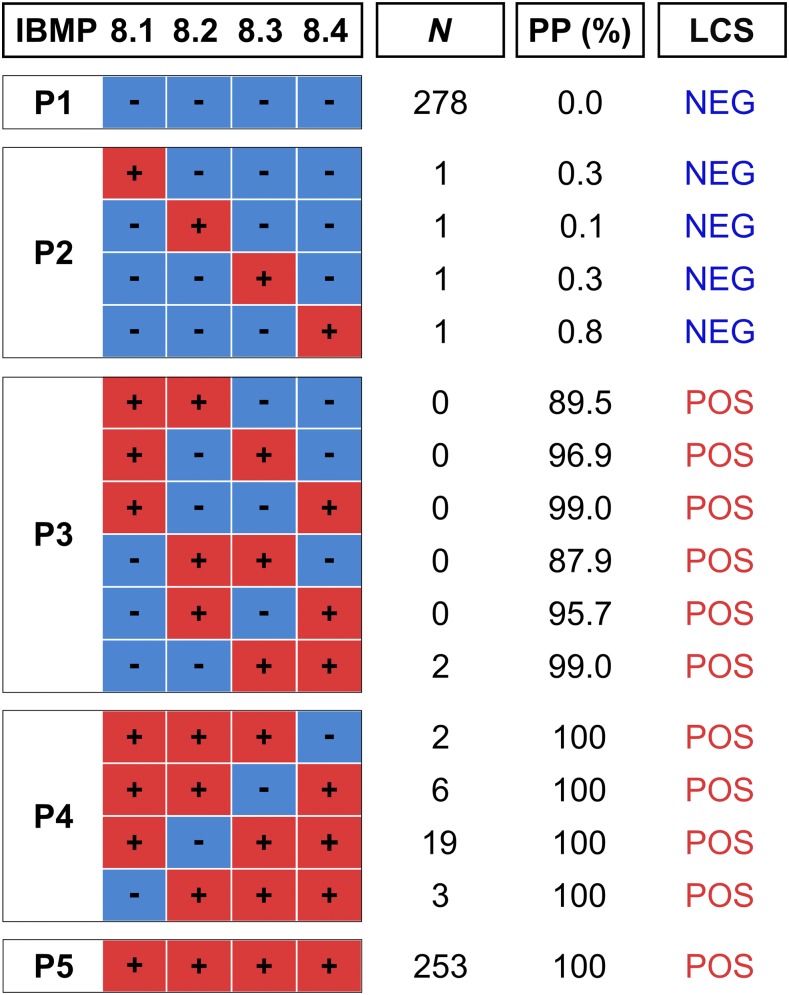
Latent class response patterns and posteriori probability of four *Trypanosoma cruzi* Molecular Biology Institute of Paraná (IBMP) chimeric antigens to accurately diagnose Chagas disease. Samples are grouped in categories P1–P5 according to the chimeric assay response pattern. Gray and black squares represent negative and positive results, respectively, for an individual IBMP chimeric antigen assay. LCS = latent class status; *N* = number of samples; NEG = negative; POS = positive; PP = posteriori probability. This figure appears in color at www.ajtmh.org.

Latent class analysis was performed using a statistical model to define a latent variable and then used as a gold standard. To define the latent variable capable of accurately identifying *T. cruzi* infection, four indicators representing IBMP-8.1, IBMP-8.2, IBMP-8.3, and IBMP-8.4 chimeric antigens were established. Sera were grouped into two categories: “positive” and “negative.” Latent class analysis is a multivariate statistical approach based on categorical indicators that express a categorical construct/latent variable. Latent classes were characterized based on the response patterns of positive/negative results from the four chimeric antigens (Figure 1) and on conditional probabilities, that is, the probability of having a particular result (positive/negative) for a chimeric antigen with respect to an individual diagnosis (positive/negative). The present LCA used maximum likelihood estimation. To evaluate the LCA model, the following criteria were used: AIC (Akaike information criteria), BIC (Bayesian information criteria), and entropy. For AIC and BIC, lower is better, whereas for entropy closest to one implies good classification quality. Conditional independence was verified using bivariate residuals. All analyses were conducted using Mplus v5.2 software (Muthén & Muthén, Los Angeles, CA). Considering the entire sample set, approximately half of each group (NCh and Ch) was randomly selected to define the gold standard used to determine *T. cruzi* infection under LCA. The other half of the sample was used to obtain estimations of sensitivity and specificity for each chimeric antigen using previously established latent class response patterns, with a corresponding CI of 95%. The area under receiver operating characteristic curves was used to estimate diagnostic accuracy, that is, to describe the capacity of the chimeric protein assay to discriminate between healthy and infected populations. These analyses were conducted using the diagt function in STATA software v12 (StataCorp., College Station, TX). *Trypanosoma cruzi* positive predictive values (PPV) and negative predictive values (NPV) were estimated with respect to a hypothetical prevalence range (from 0.05 to 0.60).

## Results

A total of 1,121 samples (526 NCh and 595 Ch) were enrolled. Of these, about 50% (283 NCh and 284 Ch) were randomly selected to estimate the response patterns and accuracy in LCA using the four *T. cruzi* chimeric antigens. The probability of each chimeric antigen to accurately predict positivity in the Ch samples was 98.2% for IBMP-8.1, 92.6% for IBMP-8.2, 97.9% for IBMP-8.3, and 99.3% for IBMP-8.4. Conversely, the probability that a given NCh sample would be classified as Ch was estimated at 0.4% for all chimeric antigens. Accordingly, the entropy value was calculated at 0.999, indicating a clear delineation among the latent class response patterns.

Figure 1 illustrates the latent class response patterns clustered according to diagnostic results obtained for the Ch and NCh samples assayed with the four IBMP chimeric antigens. Latent class response patterns were classified according to the number of positive assays: P1 (100% negative results), P2 (75% negative results), P3 (50% negative results), P4 (25% negative results), and P5 (no negative results). Despite variability in the number of samples classified in each pattern, the highest frequencies were observed in P1 and P5 categories. Samples were considered as Ch-positive when at least two chimeric antigens presented positivity (P3–P5), with posteriori probability (PP) > 87%. Conversely, samples were considered negative when no or only one IBMP tested positive (P1 and P2), with PP ≤ 0.8%.

The results of latent class status as determined by the response patterns were used as the gold standard to provide a reliable estimate of the performance of each individual chimeric assay. The IBMP-8.4 chimeric antigen yielded the highest sensitivity, achieving 99.4% (95% CI: 97.7–99.8). The sensitivity obtained for the other chimeric antigens ranged from 93.5% to 96.8% (Table 2). With respect to specificity, all chimeric antigens exhibited values greater than 99.5%, with IBMP-8.3 and IBMP-8.4 showing maximum specificity. Accuracy analysis revealed values of 96.2% for IBMP-8.2 and around 98% for IBMP-8.1 and IBMP-8.3, whereas IBMP-8.4 was found to be nearly 100% accurate (Table 2). No evidence of cross-reactivity with the evaluated leishmaniasis samples was detected.

**Table 2:** Test performance of individual IBMP chimeric antigens to diagnose chronic Chagas disease using latent class status as a gold standard

Chimeric antigen	Sensitivity (%)	Specificity (%)	Accuracy
IBMP-8.1	96.4 [93.7–98.0]	99.6 [97.7–99.9]	97.8 [96.3–98.8]
IBMP-8.2	93.5 [90.2–95.8]	99.6 [97.7–99.9]	96.2 [94.3–97.5]
IBMP-8.3	96.8 [94.1–98.2]	100.0 [98.5–100.0]	98.2 [96.7–99.0]
IBMP-8.4	99.4 [97.7–99.8]	100.0 [98.5–100.0]	99.6 [98.7–99.9]

IBMP = Molecular Biology Institute of Paraná. Values in brackets represent 95% confidence interval.

**Figure 2.**
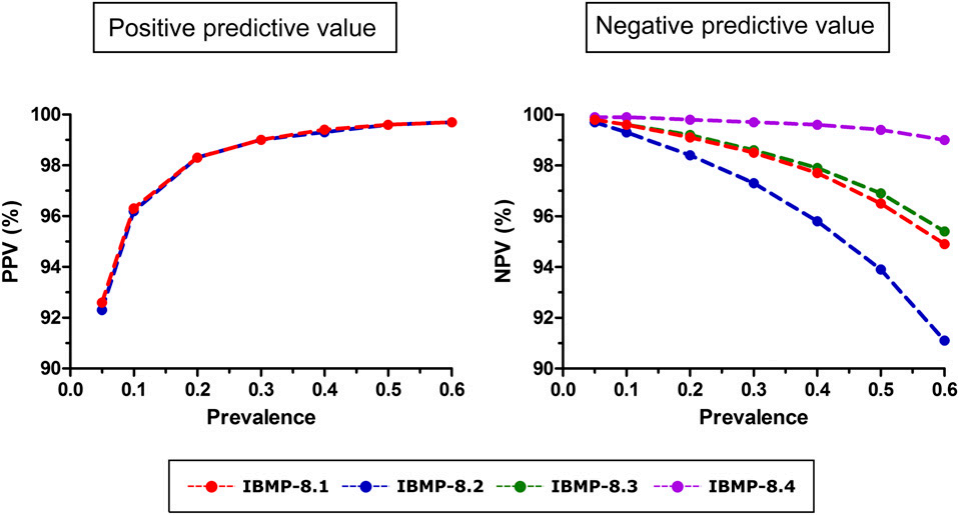
Positive and negative predictive estimates for distinct prevalence scenarios of chronic Chagas disease. IBMP = Molecular Biology Institute of Paraná; NPV = negative predictive value; PPV = positive predictive value. This figure appears in color at www.ajtmh.org.

Positive predictive values and NPV were also estimated. Because the true prevalence of chronic CD is unknown, we used a hypothetical prevalence range to evaluate distinct scenarios. Figure 2 summarizes the association between prevalence scenarios and predictive values. Increasing prevalence resulted in a correspondent change in PPV from 92.3% to 99.7% for IBMP-8.1 and IBMP-8.2. Because the specificity for IBMP-8.3 and IBMP-8.4 was 100.0%, it was not possible to estimate PPV for these chimeric antigens. A higher prevalence was correlated with lower NPV for IBMP-8.1, IBMP-8.2, and IBMP-8.3, with IBMP-8.2 (range from 99.7% to 91.1%) showing the most pronounced reduction. Molecular Biology Institute of Paraná-8.4 presented the most stable NPV, regardless of increasing prevalence.

**Figure 3.**
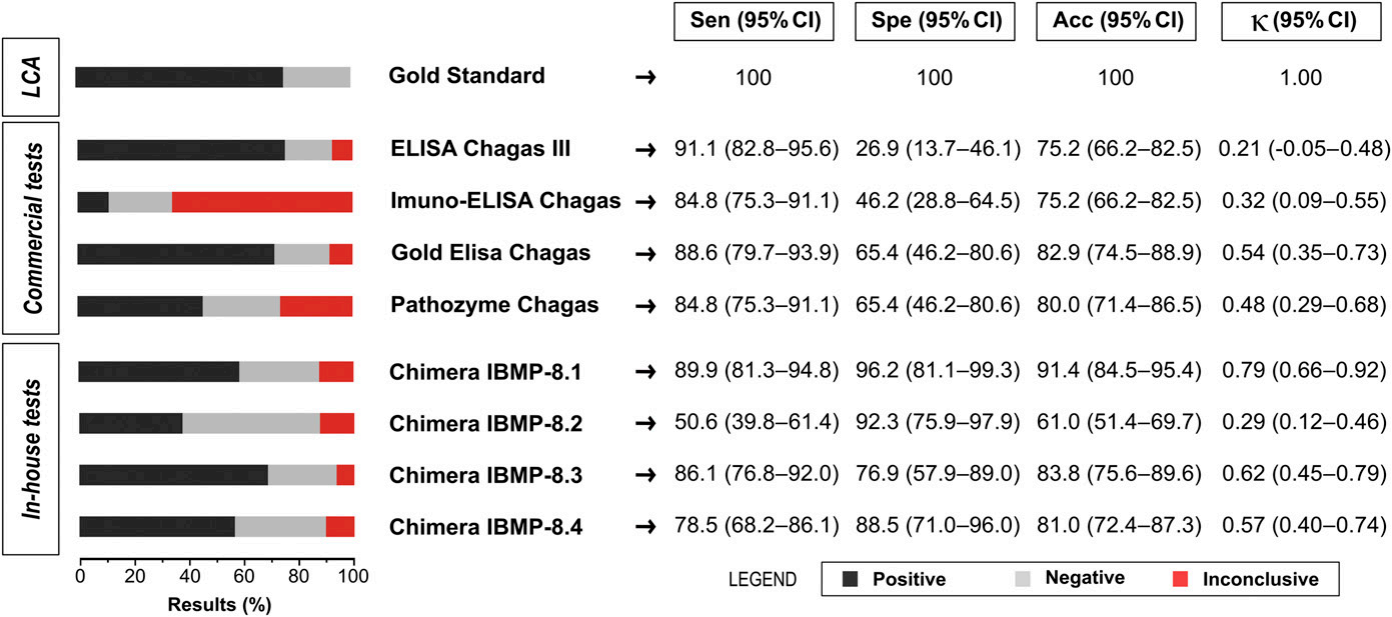
Analysis of serodiscordant samples, using LCA status as a gold standard. Acc = accuracy; CI = confidence interval; IBMP = Molecular Biology Institute of Paraná; κ = Cohen’s Kappa coefficient; LCA = latent class analysis; Sen = sensitivity; Spe = specificity. This figure appears in color at www.ajtmh.org.

A total 105 samples that had previously presented serodiscordant results under commercial testing were analyzed separately to assess the diagnostic accuracy of the four chimeras, considering the latent class response patterns described in Figure 1. The LCA model, used as a gold standard, classified 26 samples as negative (13.3% P1 and 11.4% P2) and 79 as positive (16.2% P3; 39% P4; and 20% P5). Figure 3 shows that Imuno-ELISA Chagas (65.7%) yielded the highest number of inconclusive results (represented as red bars), followed by the Pathozyme Chagas (27.6%). Sensitivity values ranged from 50.6% to 91.1%, with the lowest value obtained by IBMP-8.2, in contrast to the highest value obtained using ELISA Chagas III, followed by IBMP-8.1. Considering specificity, all IBMP chimeric antigens exhibited values greater than 80%, with the highest value achieved by IBMP-8.1 (96.2%). Conversely, all commercial tests produced specificity values inferior to 66%, with ELISA Chagas III presenting the lowest specificity overall (29.2%). The IBMP-8.1 chimera presented the highest accuracy of all diagnostic methods, which indicates its promising potential as a confirmatory test for samples with discordant results. Accuracy findings were corroborated by Cohen’s kappa, which evidenced substantial agreement (0.79) between LCA and the IBMP-8.1 chimeric antigen assay.

## Discussion

Herein a latent class model was used to estimate the diagnostic performance of four recombinant chimeric antigenic proteins to precisely detect anti–*T. cruzi* antibodies. Sensitivity and specificity values ranged from 93.5% to 99.4% and 99.6% to 100%, respectively. These results are in agreement with previous reports using commercial tests as a gold standard, in which the performance of these proteins was previously assessed by both ELISA and liquid microarray (LMA).^20,22^ With respect to ELISA, these values ranged from 94.3% to 99.3% and 99.4% to 100%, respectively,^20^ whereas under LMA, these values ranged from 96.9% to 99.1% and 99.1% to 100%,^22^ respectively. Regarding accuracy, IBMP-8.4 was found to be nearly 100% accurate (99.6%), which suggests that this antigen is the best of the four chimeras for chronic CD laboratory diagnostic purposes. On the other hand, IBMP-8.2 offered the lowest accuracy (96.2%), which is likely because of its amino acid composition, as discussed elsewhere.^20^ Intermediate values were obtained with regard to the other chimeric antigens, which are in accordance with the aforementioned studies.

Our analysis of predictive values using a hypothetical prevalence range indicated elevated PPV for IBMP-8.1 and IBMP-8.2 under increased prevalence scenarios, that is, favorable performance in an endemic context. On the contrary, decreased NPV was seen under elevated prevalence scenarios. Nonetheless, the NPV of the IBMP-8.4 antigen remained nearly constant, regardless of increases in prevalence. In response to the spread of infection worldwide, which has transformed CD into a global health concern,^25–27^ it is crucial to develop a highly accurate test for use in areas, regardless of disease prevalence, to aid in the effective clinical management of Ch patients. Accordingly, we believe that the IBMP-8.4 chimeric antigen holds great potential. Furthermore, we have already demonstrated that this particular antigen successfully recognized anti–*T. cruzi* antibodies in CD-positive individuals from distinct geographic regions in Brazil and throughout Latin America.^20^

Using four individual chimeric assays, a total of 16 response patterns were identified, which clustered into five categories. Groups P1 and P5 comprised the largest numbers of samples, which is probably because of the high accuracy offered by all four chimeras (≥ 96.6%). We observed that a negative result for all chimeric antigens (P1) or for at least three (P2) defined a given sample as NCh. Conversely, positive results for two or more chimeric antigens determined samples as Ch (P3–P5). As expected, the greatest number of samples was found in P4, specifically because of a negative result obtained by the IBMP-8.2 assay, despite positive results returned from the other chimeras. This is obviously because of the decreased sensitivity (93.5%) found for this molecule. Despite the variation seen in sensitivity, the LCA model enabled us to obtain an unbiased evaluation of the performance of IBMP chimeric antigens.

According to our results, approximately 9% of the samples were deemed inconclusive or discordant by two or more commercial tests. Imuno-ELISA Chagas yielded the highest number of inconclusive results. This kit uses recombinant proteins as antigens, which likely resulted in the high number of inconclusive cases. In fact, when this test is applied in residents of distinct geographic regions, anti–*T. cruzi* antibodies do not efficiently recognize some antigens in Ch individuals.^28^ Pathozyme Chagas also presented a high number of inconclusive results. Similar to Imuno-ELISA Chagas, this test uses recombinant proteins as antigens. The other commercial tests and the IBMP chimeric antigen assays returned a low number of inconclusive results. With regard to sensitivity, ELISA Chagas III, which uses whole extracts of *T. cruzi* strains Mn and Tulahuen as antigens, presented the highest value. Despite offering high sensitivity, this type of antigenic matrix has been demonstrated to lead to a high number of false-positive or inconclusive results,^29^ and correspondantly, this kit returned the lowest specificity value. Among the commercial tests, Gold ELISA Chagas, which uses both recombinant antigens and purified lysates from Brazilian strains of *T. cruzi* epimastigotes, was found to be the most accurate. As previously described, Gold ELISA Chagas performance was similar to that of the IBMP-8.4, IBMP-8.1 and IBMP-8.3 chimeric antigen assays.^20^ Consistent with previous findings,^20–23^ herein IBMP-8.4 was shown to offer the highest performance among the four chimeric antigens with respect to chronic CD screening and laboratory diagnostic purposes, whereas the IBMP-8.1 chimeric antigen offers the highest potential as a confirmatory test.

The present study was limited by the testing of specimens from restricted geographical origins, which represents a limited number of circulating discrete typing units, in addition to the absence of *Trypanosoma rangeli* specimens. Furthermore, the number of leishmaniasis specimens used to assess cross-reactivity was limited. However, a study using a larger sample of leishmaniasis-infected individuals (cutaneous and visceral) is currently underway. Nonetheless, our analysis confirmed the remarkable performance of these IBMP chimeric antigens in the context of chronic CD diagnosis, of which the IBMP-8.4 antigen presented superior accuracy. In addition, we call attention to the capability of the IBMP-8.1 chimera for use in confirmatory testing in cases of serodiscordant or inconclusive results.

Although the diagnostic performance of the chimeric proteins evaluated herein is promising, further investigation must be conducted to assess test accuracy in alternate scenarios, for example, of nonuniform DTUs arising from antigenic variability, in addition to comprehensively evaluating cross-reactivity with other species, including *T. rangeli* and *Leishmania* spp. In addition, as CD is considered a neglected disease, political obstacles must be overcome to improve the availability of commercial tests and more widely implement testing in not only endemic scenarios, but also routine blood testing.
